# Immunoparalysis in critical ill patients and its association to nosocomial and opportunist infections. a preliminary study

**DOI:** 10.1186/2197-425X-3-S1-A1016

**Published:** 2015-10-01

**Authors:** E Villarreal, P Ramirez, L Cordon, P Geffner, A Cortes, M Gordon, E Gonzalez, P Kot, J Ruis, A Sampere

**Affiliations:** Hospital La Fe, Valencia, Spain

## Introduction

There is a growing molecular and clinical evidence of immune impairment or immunoparalysis (IP)in critically ill patients.IP could be a physiological attempt to return to homeostasis after an intense pro-inflammatory phase and would explain the prevalence of nosocomial and opportunist infections,in critically ill patients initially considered as an immunocompetent patients.

## Objectives

1. Evaluate the immune status of immunocompetent critically ill patients at admission, and on day 3 and 7.

2. Analyze the relationship between the immune status of the critically ill patient and the risk of nosocomial or oportunist infections.

## Methods

Prospective and observational study.We included patients older than 18 years old, admitted to ICU and undergoing mechanical ventilation, who have been presented an acute failure of unless two organs.

At admission, and on day 3 and 7, we determined:

- Immunophenotypic lymphocyte subpopulations (ILS) (T, B and NK), concentration of immunoglobulins in serum, and expression of HLA-DR on monocytes and FOXP3 on T cells.

- Mini-bronchoalveolar lavage for bacterial, viral and fungal culture, detection by polymerase chain reaction of cytomegalovirus, herpes simplex virus and *Aspergillus* spp, and determination of galactoman.

- Serum sample to detect immunoglobulins (IgM and IgG) against HSV and CMV, and quantitative measurement of CMV DNA.In those patients diagnosed and treated for nosocomial or opportunist infection, we evaluated the same variables, the day of infection and after 72 h.

All results are expressed as the median with interquiartile. Statistical analysis was performed using the Mann-Withney U test or kruskall-Wallis analysis. Data will be processed with SPSS 16.0.

## Results

23 patients were included.The study population contained 77,3% male, with a median age of 59 years. Baseline severity of illness was quantified using the SAPSIII score, the median was 63. The main reason for admission was cerebrovascular accident 31,8% and sepsis (13,6%). Mortality rate was 59%.At admission, levels of different ILS, concentration of immunoglobulins in serum and expression of HLADR were lower than those found in subsequent tests, without statistically significant differences. Six patients were diagnosed of a nosocomial infection (83% ventilator associated pneumonia) and one was diagnosed of an oportunist infection (invasive pulmonary aspergillosis).

The deceased patients with a nosocomial infection, when they were diagnosed, had lower levels of ILS immunoglobulins, and expression of HLADR and FOXP3 than survivors, without statistically significant differences (table 1).

**Table 1:** Patients with nosocomial infection.

	Survivors	Deceased
CD3	1237 [185-1243]	911,5 [740-1083]
CD8	446 [52-689]	376,5 [279-454]
B	279 [58-663]	42 [20-64]
NK	84 [15-132]	63 [54-72]
IgA	353 [133-450]	226 [160-293]
IgG	1080 [973-1660]	671 [660-683]
IgM	123 [36-145]	78 [51-105]
TregsFoxP3+ (% T cell)	44,5 [39,9-49,12]	29,4 [4,96-38,8]
HLADR+ (% PMN)	52,8 [50,6-55]	40,03 [27,04-85,33]

## Conclusions

At admission in intensive care unit there was an immunosuppression.Patients with nosocomial and opportunist infection, show a lack of immune recovery at the immune monitoring. This immunosuppression is more marked in deceased patients and therefore could be correlated with an increase of mortality.

